# Identifying the genomic determinants of aging and longevity in human population studies: Progress and challenges

**DOI:** 10.1002/bies.201200148

**Published:** 2013-02-19

**Authors:** Joris Deelen, Marian Beekman, Miriam Capri, Claudio Franceschi, P Eline Slagboom

**Affiliations:** 1) Section of Molecular Epidemiology, Leiden University Medical CenterLeiden, The Netherlands; 2) Netherlands Consortium for Healthy Ageing, Leiden University Medical CenterLeiden, The Netherlands; 3) Department of Specialized, Diagnostic and Experimental Medicine, University of BolognaBologna, Italy

**Keywords:** biomarkers, genomics, healthy aging, human, longevity

## Abstract

Human lifespan variation is mainly determined by environmental factors, whereas the genetic contribution is 25–30% and expected to be polygenic. Two complementary fields go hand in hand in order to unravel the mechanisms of biological aging: genomic and biomarker research. Explorative and candidate gene studies of the human genome by genetic, transcriptomic, and epigenomic approaches have resulted in the identification of a limited number of interesting positive linkage regions, genes, and pathways that contribute to lifespan variation. The possibilities to further exploit these findings are rapidly increasing through the use of novel technologies, such as next-generation sequencing. Genomic research is progressively being integrated with biomarker studies on aging, including the application of (noninvasive) deep phenotyping and omics data – generated using novel technologies – in a wealth of studies in human populations. Hence, these studies may assist in obtaining a more holistic perspective on the role of the genome in aging and lifespan regulation.

## Introduction

Human life expectancy has increased remarkably over the last two centuries worldwide 1, although it is still highly variable between countries 2. This lifespan extension is mainly due to improvement of health care, hygiene, and nutrition. The healthy life expectancy, however, has not increased at the same rate; in Europe, men spend on average 20.5% and women 25.4% of their life dealing with disability caused through disease or injury (Healthy Life Years; http://www.healthy-life-years.eu/) 3. Although age is the main risk factor for the majority of common diseases contributing to disability, reaching an old age does not necessarily result in a higher degree of age-related disability. This is illustrated by the presence of long-lived individuals from families expressing exceptional longevity that may reach high ages without major disabilities 4, 5. Moreover, their offspring – considered “decelerated” or “healthy agers” – have a lower prevalence of age-related diseases, such as cancer, cardiovascular disease, hypertension, and type 2 diabetes 6–9, compared to similar-aged controls. Concomitantly, they show beneficial or “youthful” profiles for many metabolic and immune-related parameters 10. Most of the human aging studies are concentrated around long-lived families, including highly and middle-aged members, sporadic highly aged individuals from the general population or population-based cohorts containing different age groups.

Due to the different study designs (Box 1 and Fig. 1), human aging cohorts provide complementary information and are intensively being studied from a biomarker and genomic perspective. The assumption is that, together, these studies will provide insight into the mechanisms that could (i) drive the biological aging rate, (ii) positively and negatively influence the risk for age-related disease, and (iii) explain the variation in lifespan between individuals. Genomic research, including genetic, epigenetic, and transcriptomic studies, is expected to provide both markers and determinants of aging. The search for biomarkers for human aging and longevity is aimed at identifying parameters and profiles that reflect the biological age of individuals and predict long-term morbidity and/or mortality 11.

**Study designs**

The ultimate epidemiological study design to investigate markers and determinants of biological aging and longevity in humans would be to follow a large group of individuals during their entire lifetime. These individuals should be examined at different time points so that changes in markers could be related to the actual lifespan of the individual. However, since this design is not feasible, several other designs are being applied in human studies (Fig. 1).

**Cross-sectional study designs**

***Population-based cohorts*:** Cross-sectional longevity studies typically compare unrelated highly aged individuals (nonagenarians/centenarians) with younger controls or evaluate differences between groups of unrelated individuals in categories of increasing age. Inclusion of individuals for these studies is relatively easy, which is reflected by the large sample sizes of population-based cross-sectional studies. The cross-sectional study usually provides the first level of observation that a parameter is correlated with chronological age or a health condition. However, causality of the genetic and/or genomic parameter on aging and longevity cannot be determined from a cross-sectional design. For cross-sectional studies the long-lived cases should be compared with controls originating from the same birth cohort. However, since these controls usually already died, controls are generally selected from other birth cohorts. Given that these cohorts have a different life expectancy, this could confound the studied association. In addition, structural differences between birth cohorts, caused by, e.g. migration, could also confound the results. Examples of longevity studies used for cross-sectional analysis in unrelated individuals are the New England Centenarian Study (NECS) 90, German long-lived individuals 91, French centenarians 92, and Southern Italian Centenarian Study (SICS) 93. In addition, various cross-sectional studies are included in the MARK-AGE project, which consists of 2,320 randomly recruited volunteers from the general population (35–74 years).

***Family-based cohorts***: Family-based longevity studies consist of nonagenarians/centenarians (siblings) and their middle-aged offspring. The controls used in these studies are either (age-matched) random individuals from the general population or spouses of the offspring of the long-lived individuals. Due to the common genetic background among family members, family-based longevity studies are enriched for familial and genetic effects on longevity and are more robust against population substructure. However, these studies generally have a small sample size since it is quite difficult to collect long-lived families. To determine which age-related phenotypes associate with human familial longevity, the offspring of long-lived individuals, which are predisposed to longevity, can be compared to geographically- and age-matched population controls. This design allows analysis of molecular and clinical parameters specific for long-lived family members in multiple generations. Examples of family-based longevity studies are the Ashkenazi Jews cohort 71, GEnetics of Healthy Ageing (GEHA) study (of which the offspring is collected in the MARK-AGE project) 94, Long Life Family Study (LLFS) 7, and Leiden Longevity Study (LLS) 15.

**Prospective studies**

Most prospective longevity studies consist of highly (>85 years of age) or middle-aged (>55 years of age) individuals (related or unrelated) that are followed over time and sampled at multiple time points. This design is most often applied to provide more evidence for causality of determinants or markers detected in cross-sectional studies. In this design an (unbiased) baseline parameter may show to precede a functional aspect of aging. Several large population-based prospective studies have been initiated. However, the main disadvantage of these studies is that the number of individuals that will become long-lived is usually very small. Examples of prospective longevity studies are the Leiden 85-plus study 95, 96, Newcastle 85+ study 97, 98, Danish 1905 cohort 99, the population-based Rotterdam Study 100, and Framingham Heart Study (FHS) (consisting of three generations) 101.

**1:**
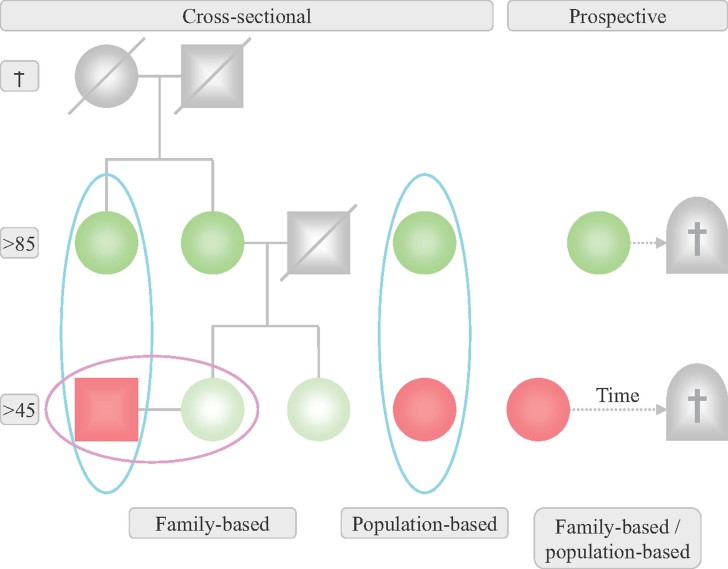
Study designs applied in studies of healthy aging and longevity. Family- or population-based cross-sectional designs usually compare highly aged individuals with younger controls (blue ovals). Alternatively, the offspring of long-lived individuals is compared to age-matched controls (their spouses or random population controls) (purple oval). Thirdly, prospective studies are performed in highly or middle-aged individuals (unrelated or from (long-lived) families) which are followed over time (ranging from 10 to 30 years, depending on the study). Highly aged individuals are depicted in green, their offspring in light green and middle-aged individuals in red.

For most diseases, like osteoarthritis, osteoporosis, and type 2 diabetes, standardized phenotypes and diagnostic criteria are used for genomic research. No standardized phenotype or marker, however, is indicating biological aging rate. Hence, genomic studies into aging thus far focus on the determinants of human lifespan variation by using age at death, prospective survival, disease-free survival, or exceptional longevity as outcome. Biomarker research is therefore just as relevant for genomic studies of human aging as the analysis of the genome itself.

The possibility to study causal determinants and quantitative biomarkers of biological aging and longevity in humans strongly depends on the study designs that are available (Box 1 and Fig. 1). Using these designs, we determined four relevant phases in aging studies in order to establish whether a quantitative parameter (or profile) is a biomarker of biological age; (i) Determine the change in a quantitative parameter with chronological age in cross-sectional studies and, preferably, by repeated measures in longitudinal studies. Parameters reflecting biological age are expected to show an increased variance with age. (ii) Determine whether a marker of chronological age also discriminates individuals with a youthful or old level relative to their age category in the general population, which would indicate that the quantitative parameter potentially marks biological age (Fig. 2). The comparison between offspring of long-lived individuals and age-matched population controls is also part of this phase. (iii) Determine whether the potential marker for biological age associates to known parameters of health, such as blood pressure, serum levels of glucose, insulin, and cholesterol. (iv) Determine whether the potential marker for biological age associates with morbidity (based on clinical endpoints) and/or mortality in prospective studies.

**2:**
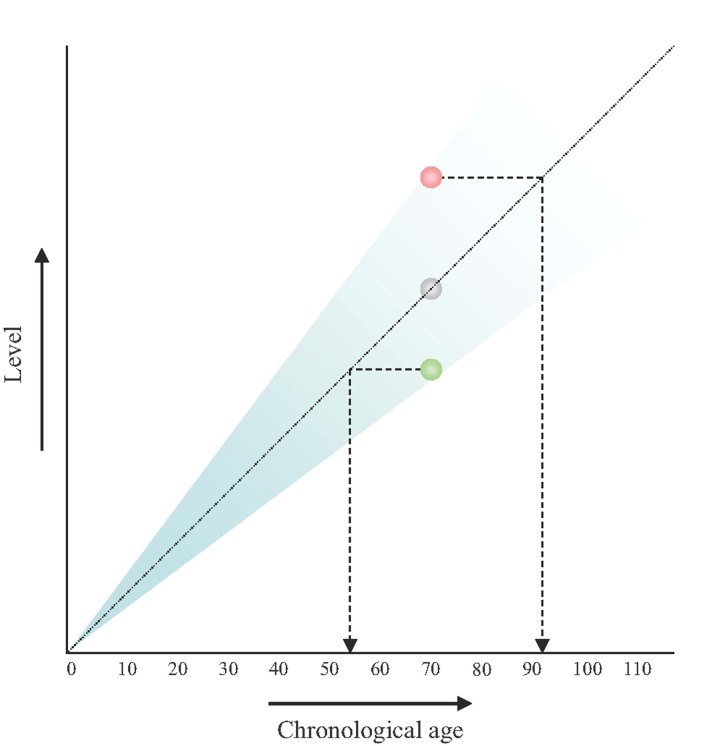
Interpretation of the potential relationship between a marker of chronological age and biological age using categories of increasing age. The blue zone indicates the increasing variance of the marker with age. Individuals can be assigned to having a marker level which matches (i) the expected level for their age in the population (gray dot, 75 years in this example), (ii) the level of a younger age group (green dot, biological age may be lower than chronological age), or (iii) the level of an older age group (red dot, biological age may be higher than chronological age).

In this review we will give an overview of the main genomic approaches and discuss the concept of biomarker approaches used in the research field of human aging and longevity. In addition, we will discuss the progress and challenges of integration of data that has been generated using these approaches.

## Genomic research

### Human longevity is not just explained by the absence of disease-susceptibility alleles

Genomic research into human lifespan regulation could be subdivided into genetic, epigenetic, and transcriptomic research. Studies of mono- and dizygous twins have revealed that the genetic contribution to the variation in human lifespan is about 25–30% 12, 13, and is most prominent in families clustered for longevity 14, 15. This genetic contribution is mainly apparent after the age of 60 years and seems to increase with age 13, 16. Furthermore, human lifespan is a complex trait which is assumed to be determined by many genes with small individual effects 17, although the polygenic architecture still needs to be characterized 18, 19. The diverse health features of long-lived families illustrate that different age-related diseases have common determinants and implicate that pathways can be identified that attenuate aging and delay age-related disease. From a genomic perspective, individuals from long-lived families are assumed to be characterized by a decreased prevalence of disease-promoting variants (referred to as disease-susceptibility alleles) and an increased prevalence of variants conferring maintenance of health and protection from disease, when compared to population controls. In the last 5 years, many disease-susceptibility alleles have been identified (National Human Genome Research Institute (NHGRI) genome-wide association study (GWAS) Catalog; http://www.genome.gov/gwastudies/) 20. A first comparison between long-lived individuals, selected from both long-lived families (LLS) and the general population (Leiden 85-plus study), and young controls showed no difference in the distribution or frequency of disease-susceptibility alleles identified in cancer, coronary artery disease and type 2 diabetes 21. The search for lifespan regulating loci – contributing to longevity and population mortality – must therefore extend beyond a focus on disease-susceptibility alleles. We will first discuss the efforts to identify longevity loci by genetics approaches.

### Candidate gene studies identified *APOE* and *FOXO3A* as human longevity genes

The first genetic longevity studies mainly focused on lifespan regulating loci that emerged from animal models 22. Lifespan extension in animal models was obtained by applying caloric restriction or by modifying gene functions (mutagenesis) using RNA interference, knock-out or overexpression of single genes (GenAge; http://genomics.senescence.info/genes/) 23. The most interesting pathways identified using these models are the growth hormone (GH)/insulin/insulin-like growth factor 1 (IGF-1) signaling and mammalian target of rapamycin (mTOR) signaling pathways 24. Thus far, lifespan has been the main phenotype investigated in animal models. In order to make these models more translatable to human studies research should focus on defining the parameters that reflect the physiology and pathology of aging in both animals and humans 25, 26.

Most of the human candidate gene studies were performed in cross-sectional designs (Box 1 and Fig. 1), comparing allele frequencies of potential longevity loci between highly aged individuals and young controls. The candidate gene studies based on single genes have pointed a role for genes involved in, e.g., GH/insulin/IGF-1 signaling, immune regulation, and lipoprotein metabolism (Supporting Information Table S1), although most of these results have not (yet) been confirmed in sufficient independent studies. The most convincing human longevity loci today are *APOE* and *FOXO3A* which have frequently been associated with longevity in cross-sectional studies (see for a review 26) and survival in prospective studies 27–29 (Fig. 3). *APOE* encodes the protein apolipoprotein E which seems to play a role in e.g., lipoprotein metabolism, cognitive function, and immune regulation 30. *FOXO3A* encodes the protein forkhead box O3 which acts as a transcription factor for many different genes involved in processes like apoptosis and oxidative stress 31.

**3:**
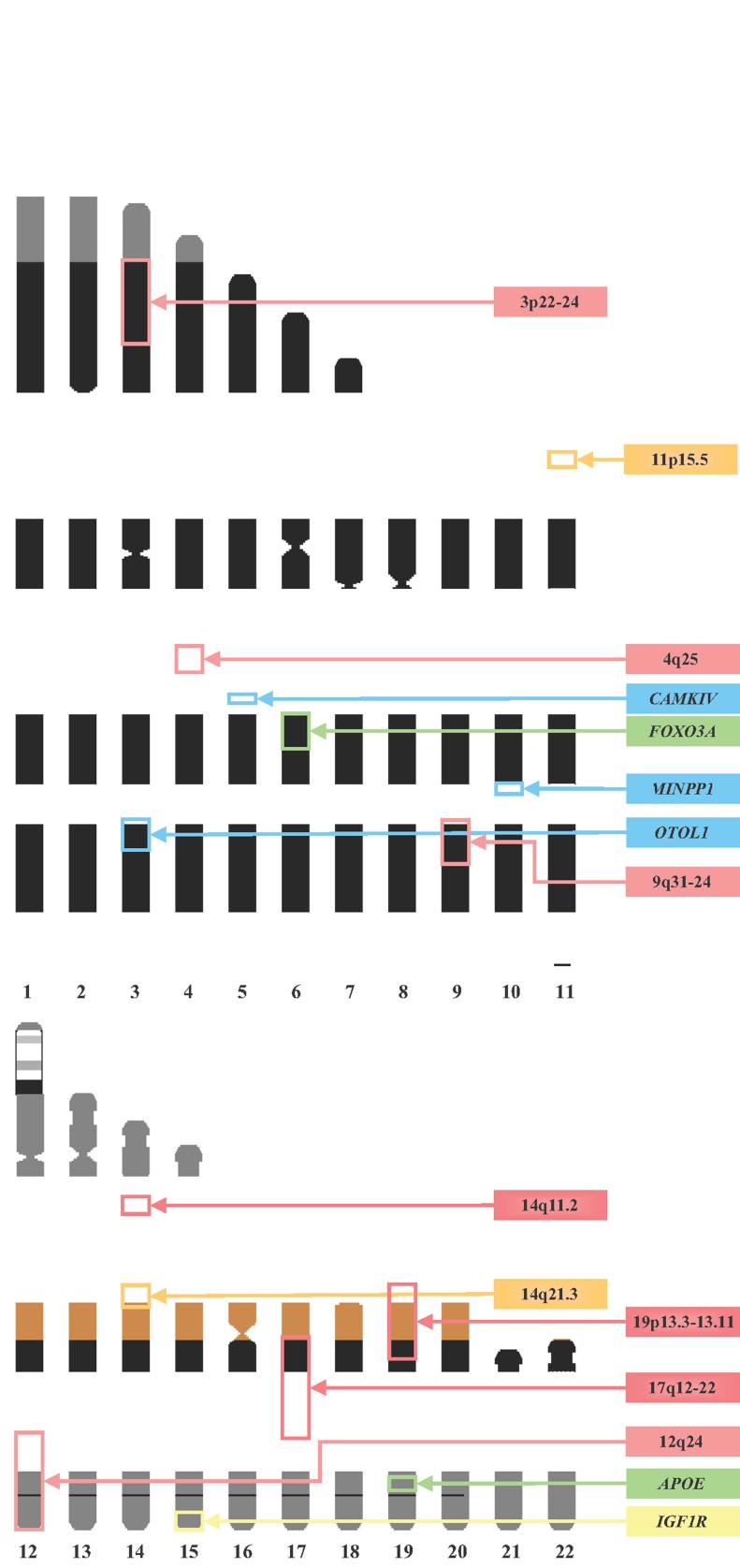
Karyogram (adapted from http://hapmap.ncbi.nlm.nih.gov/karyogram/gwas.html) containing candidate genes whose association with longevity has been replicated in multiple association studies (green), candidate genes with interesting results from sequencing studies (yellow), interesting loci from linkage (LOD ≥ 2.95) (red), and CNV (orange) studies and loci that showed suggestive association with longevity (*p* ≤ 5 × 10^−6^) in GWAS (blue).

In addition to single gene studies, several candidate gene studies based on whole pathways have been performed. These pathway-based candidate gene studies showed a role for genes within the DNA damage signaling and repair, GH/insulin/IGF-1 signaling, immune regulation, pro/antioxidant, and telomere maintenance pathways 32–36 (Supporting Information Table S1). Most of these pathway-based studies tested for effects of individual single nucleotide polymorphisms (SNPs) on prospective mortality or longevity 32, 34, 35, and, so far, only a limited number of studies determined the joint effect of SNPs within a pathway 33, 36.

### Large meta-GWAS are required for identification of novel human longevity loci

As an alternative to hypothesis-based candidate gene studies, hypothesis-free or explorative approaches could also be applied to studies of the genome. These methods should initially be aimed at prioritizing the location of regions linked to longevity and, subsequently, identifying the genetic variation causal to the trait. One example of an explorative approach is the GWAS. In this cross-sectional approach, in which long-lived individuals are compared with young or shorter-lived controls, the-usually small-effect of common variants can be identified. Typically, genotype distributions of 300,000–2,500,000 SNPs are assessed for association with the trait in GWAS. Since longevity is assumed to be determined by many genes with small effects it could be a successful method to identify novel longevity loci. However, so far, GWAS for longevity in the LLS 37, Cohorts for Heart and Aging Research in Genomic Epidemiology (CHARGE) 38, 39, NECS 40, German long-lived individuals 41, and SICS 42 have only identified one genome-wide significant (*p* < 5 × 10^−8^) locus: *APOE*, which has long been established as a longevity gene. Several other loci showed suggestive association with longevity (*p* < 5 × 10^−6^), namely *MINPP1* 38, *OTOL1* 39, and *CAMKIV* 42 (Fig. 3).

However, the effect of these loci on prospective mortality is not yet known. All GWAS-identified suggestive longevity loci are deleterious, i.e., the minor allele is associated with a decreased probability to become long-lived, and, as expected, their effects are small (odds ratio's > 0.5).

In general, to have sufficient power to detect significant effects, GWAS require much larger sample sizes than thus far accomplished for human longevity. One of the challenges of GWAS for longevity is that the lifespan variation induced by the genetic component is expected to be small relative to that induced by the environmental component (i.e., health care and nutrition). A large sample size, acquired through metaanalysis of GWAS (meta-GWAS), may cope with the so-called “phenocopies” and could potentially detect genome-wide significant loci besides *APOE*. Currently, two initiatives for meta-GWAS for longevity are on-going. One consists of ∼8,000 long-lived individuals (≥85 years of age) from all over Europe (Integrated research on DEvelopmental determinants of Aging and Longevity (IDEAL) GWAS Longevity Study), while the other consists of ∼6,000 long-lived individuals (≥90 years of age), collected in Northern America and Europe, from the CHARGE consortium. If these meta-GWAS lead to the identification of new loci that significantly associate with longevity, they should consequently be tested for an effect on prospective survival in middle and old age.

### Copy number variant (CNV) studies identified potential longevity regions

Besides SNP analysis, several other methods have been applied to study the genetics of longevity, mainly using a prospective design (Box 1 and Fig. 1). One study determined the effect of CNVs, which are deletions or duplications of stretches of DNA, on longevity in the Rotterdam Study and FHS. The meta-analysis of these cohorts showed an association between the burden of large (≥500 kb) CNVs and mortality at old age. In addition, they showed an association of common CNV regions on 11p15.5 and 14q21.3 43 (Fig. 3). However, to qualify them as longevity-regions, these associations still need to be replicated in several larger independent cross-sectional and prospective studies.

The same group also studied the effect of regions of homozygosity (ROHs), which are uninterrupted stretches of homozygous SNPs, on longevity in the Rotterdam Study and found no association between ROHs and survival into old age 44. However, to rule out effects of ROHs on longevity larger cross-sectional and prospective studies should be performed.

### Linkage studies have discovered chromosomal regions linked to human longevity

The explorative studies of the genome for longevity effects actually started with linkage analysis in family-based designs (Box 1 and Fig. 1). For this approach, the excess sharing of alleles between siblings identical by descent at 6,000–12,000 loci not in linkage disequilibrium over sharing by chance provides a likelihood for the presence of a longevity locus in any region on the genome. There have been several small-scale genome-wide linkage studies of long-lived sibling pairs (*n*_cases_ < 300) that showed inconsistent results 45–48 (Fig. 3). Recently, a large linkage analysis for longevity has been performed in 2,118 nonagenarian Caucasian sibling pairs from the GEHA study. In this study, linkage with longevity was observed at chromosome 14q11.2 (logarithm (base 10) of odds (LOD) = 3.47), chromosome 17q12-22 (LOD = 2.95), chromosome 19p13.3-13.11 (LOD = 3.76), and chromosome 19q13.11-13.32 (LOD = 3.57) (Fig. 3), of which the latter was explained by the ApoE ε4 and ApoE ε2 alleles 49. Since the linkage at the remaining loci could not be explained by association of common variants, human familial longevity at these loci may be explained by rare variants.

### Next-generation sequencing studies may reveal rare longevity-associated variants

Rare variants can be identified by applying next-generation (whole-genome or exome) sequencing. In the case of Mendelian disorders and strong familial traits, next-generation sequencing of a limited number of well-selected individuals may reveal relevant alleles with functional consequences. Analysis of sequencing data is a bioinformatic challenge and good sample selection is therefore extremely important. The most informative individuals for next-generation sequencing in longevity research would be individuals from long-lived families with a long family history of longevity. One candidate gene study analyzed the complete coding region of *IGF1* and *IGF1R* using 2D gene scanning and DNA sequencing in centenarians and their offspring. Two rare nonsynonymous SNPs in *IGF1R* associated with both longevity and decreased IGF-1 signaling. This further indicates a role for GH/insulin/IGF-1 signaling genes in human longevity 50 (Fig. 3).

For exploratory analyses, the whole genome can be analyzed. Up to now, this has been published for one female and one male supercentenarian 51. To identify variants relevant for longevity, analysis on the genomes of many more of such individuals must be performed. Various initiatives are ongoing in which larger amounts of genomes of population and family-based centenarians are being sequenced, e.g., the Wellderly Study (consisting of ∼1,000 individuals ≥80 years of age) and the LLS (consisting of 220 individuals ≥90 years of age).

### Explorative studies identify transcriptomic profiles marking longevity

Since the genetic approaches have thus far provided little robust evidence for loci contributing to human aging and longevity, attempts have been made to identify such loci by exploration of the human transcriptome. The transcriptome of an individual reflects the influence of genetic variation, as well as the response to the environment. As an approach to find determinants of aging and longevity, transcriptomic studies require specific designs to disentangle primary and causal changes in gene expression from the consequences of aging.

Most studies of the transcriptome try to identify genes that show a differential change with chronological age and mainly use cross-sectional designs (Box 1 and Fig. 1). In these designs highly aged individuals are compared to young controls or categories of increasing age are examined. The larger studies are performed in whole blood, since this is the most accessible tissue. However, whole blood contains different cell populations, which may confound observed differences in gene expression. If possible, observations of differential gene expression should thus be adjusted for proportions of blood cell subsets, which is not always done. One study partly circumvented this problem by investigating the transcriptome of T cells from healthy individuals with ages ranging from 25 to over 95 years and highlighted similarities in gene expression profiles between young and “successfully aged” individuals 52. This illustrates that cross-sectional transciptome studies may be used to identify genes potentially indicative of the biological age of an individual by comparing the expression level of the gene for an individual to the average expression of individuals of his/her chronological age.

The transciptomic studies focused on chronological age revealed that genes and microRNAs involved in many different processes, e.g., oxidative phosphorylation, complement activation, and synaptic transmission, change with age 53–58. The pathways that have been associated with chronological age include peroxisome proliferator-activated receptor, glucose and glutathione metabolism, and mTOR signaling 52. The relevance of mTOR pathway genes for human aging has been further illustrated by associations of gene expression changes with chronological age in a candidate gene study of two independent human cohorts 56. Most of the gene expression associations with chronological age in human populations have not yet been validated and replicated with comparable technology platforms in independent studies. In addition, transciptomic studies on chronological age cannot rate which changes are causal and which are consequential to aging.

One way to overcome (part of) this problem is by using a family-based study design (Box 1 and Fig. 1), in which the offspring of long-lived individuals – representing “healthy agers” – are compared to similar-aged controls from the general population. The differential gene expression profiles identified using this design may represent markers of healthy aging and familial longevity. This approach has been applied in the LLS to explore the transcriptome in whole blood for association with human familial longevity. Genes belonging to the mTOR pathway, as well as *ASF1A* and *IL7R*, were differentially expressed between offspring and controls 59, 60. In addition, the expression of mTOR genes in blood associated to prevalent diabetes and serum glucose. However, the association with familial longevity was not dependent on this. Thus, gene expression profiles in blood mark human longevity in middle age and potentially provide information on the pathways that contribute to healthy aging and longevity.

### Epigenomic studies are at hand

Another molecular level that could provide additional insight in the processes of aging is the epigenome, the intermediate layer of genomic information between the genome and transcriptome. Epigenetic regulation of transcription is mediated by histon modification, DNA methylation, and microRNAs. Changes in the epigenome with chronological age have been explored and show that methylation patterns of genes involved in e.g., development and morphogenesis, DNA binding and regulation of transcription 61–63 tend to change with age. A recent remarkable finding in a small study sample, confirmed in a cohort of 501 individuals ranging from birth to 99 years, was the progressive linear increase in methylation with age at the *ELOVL2* gene 64. Because the epigenomic field recently became more accessible for the screening of large study populations, the identification of a new range of epigenetic biomarkers is at hand. To consider such epigenetic measures as markers for biological age, confounding of cell type distributions should be accounted for – like in transcriptomic studies – and effects should be established using various study designs.

In conclusion, up to now, genomic research to identify drivers of healthy aging and longevity in humans has not yet delivered many robust longevity loci and pathways. However, larger studies, new methodologies and the consistent use of different study designs to follow up results might help to unravel the genomic component of healthy aging and longevity.

## Phenotypes that reflect biological aging

In addition to focusing on lifespan as primary phenotype, genomic studies into aging may profit from insights into phenotypes that reflect biological age. One can think of parameters or profiles reflecting immunosenescence or metabolic health established as pre-clinical measures in middle-aged individuals. In addition, phenotyping by novel noninvasive technologies, such as imaging (e.g., functional magnetic resonance imaging (MRI)) and longitudinal and ambulatory measurements using electronic devices (e.g., gait speed, 24-hour glucose, blood pressure), will improve the monitoring of the physiology of aging in epidemiological studies. Such research is often referred to as biomarker research and is aimed at finding parameters and profiles predicting long-term morbidity and/or mortality. Classical examples are blood pressure and hypertension as markers for clinical events in cardiovascular disease, joint-space width as marker for osteoarthritis and bone mineral density and risk of fracture as markers for osteoporosis. Comparable to the genomic research of the transcriptome and epigenome, the main problem with biomarker research is that it is hard to disentangle the changes causal to aging and longevity from those that are a consequence of normative aging. For classical (e.g., leukocyte telomere length) and novel potential biomarker of aging the four relevant phases to establish whether a quantitative parameter (or profile) is a biomarker of biological aging should be taken into account (see Introduction section).

### Clinical biomarkers for biological age hint at metabolic processes

Several prospective studies investigated the effect of clinical, physical, and cognitive parameters on mortality. Many different parameters have been shown to influence mortality after 55 years of age in the general population 65–70. To determine whether these parameters potentially contribute to longevity from middle age onwards, family-based studies have been performed (Box 1 and Fig. 1), whereby the offspring of long-lived individuals is compared with similar-aged controls from the general population. Of the parameters that associate with mortality after 55 years of age, cortisol levels, digit symbol substitution test score, fasting glucose levels, free triiodothyronine levels, and gait speed also mark familial longevity in middle age 7, 71–78 (Table 1). Together, these biomarkers for biological age suggest the involvement of metabolic processes in healthy aging and longevity.

**Table 1:** Potential biomarkers which have been shown to be associated with mortality in prospective studies of different age categories and their association with familial longevity in middle age

Parameter	Effect on mortality	Reference	Effect on familial longevity	Reference
Ankle-arm index	>65 years	66	No	7
Blood pressure (diastolic)	>55 years/>90 years	65, 69, 70	No	7
Blood pressure (systolic)	>55 years/>90 years	65, 66, 69, 70	No	7
Body mass index	>55 years/>90 years	69, 70	No	7, 77
Cholesterol (high-density lipoprotein)	>85 years	65, 69, 70	No	7, 71, 77
Cholesterol (total)	>55 years/>90 years	65, 69, 70	No	7, 71, 77
Cortisol	>85 years	65	Yes	73
C-reactive protein	>55 years	65, 66, 70	No	76
Creatinine	>55 years/>65 years	65, 66, 70	No	7, 78
Digit symbol substitution test score	>65 years	66	Yes	7
Fasting glucose	>65 years	65, 66	Yes	7, 74
Forced vital capacity	>65 years	66	No	7
Free triiodothyronine levels	>85 years	65	Yes	75
Gait speed	>65 years	68	Yes	7
Grip strength	>92 years	65, 67	No	7, 72
Instrumental activities of daily living impairment	>65 years	66, 70	No	7

### Metabolic profiles seem promising predictive biomarkers

Instead of testing single quantitative parameters from a clinical perspective, the development of novel technologies and methodologies has made it possible to study age-related changes in the whole glycome and metabolome 79, 80. These novel explorative omics studies could potentially be much more informative on physiological aspects of aging than the single parameters studied so far, since a single-point measurement contains a wealth of information. A cross-sectional comparison of “healthy agers” and similar-aged controls has shown that decreased levels of bisecting GlcNAc glycoforms of IgG and higher levels of specific *N*-glycan features mark healthy aging and familial longevity 81, 82. Datasets generated by metabolomic platforms provide information on biogenic amines, central metabolism, and lipids and can give insight into their relevance for morbidity and/or mortality, as was previously shown for cardiovascular disease 83. In a recent study, using a prospective design, it was shown that a single-point nuclear magnetic resonance (NMR) measurement could also predict incident risk of coronary heart disease, comparable to the gold standard (the Framingham risk score) (unpublished results). However, additional prospective studies into morbidity and/or mortality, preferably on the basis of repeated measures, need to be performed to provide more information about the usefulness of metabolomic and glycomic profiles as biomarkers of biological age and longevity.

### Integrating genomics and biomarker research

Once the use of established biomarkers of biological age is standardized, the biomarker information can be integrated into studies aimed at finding causal determinants of aging and longevity. An example of an integrated approach to identify lifespan regulating loci is represented by testing whether genetic variants associated with potential biomarkers also associate with longevity. To date, GWAS have identified many genetic variants that associate with age-associated traits, such as leukocyte telomere length and features from glycome and metabolome profiles 84–86. The joint effect of the majority of these variants on aging and longevity still needs to be determined. One study identified a haplotype in the *TERT* gene that was associated with increased telomere length and longevity, which indicates that genetic variants associated with telomere length regulation might also play a role in longevity 87.

## Conclusions and prospects

Over the past two decades the human aging field has built up the necessary resources to study the biology of aging and longevity by establishing human populations with a diversity of designs. Meta-analyses integrating genetic and phenotypic datasets have successfully identified variants associated with a range of age-related traits and diseases. Despite these accomplishments, the number of novel leads contributing to human lifespan regulation is limited. Although positive regions of linkage and suggestive GWAS hits have been reported, the field has not yet identified the loci that explain the clustering of longevity in families and the variation in biological aging rate in the population. As for animal models, down-signaling of the IIS and mTOR pathway appeared to be relevant in humans. These findings are being followed up by molecular and physiological profiling using skin, fat and muscle tissue of long-lived family members and controls. Human studies now also include the response of nutrient sensing systems to the application of dietary and physical challenges.

The ongoing whole genome sequencing of centenarians and their families may provide novel genes contributing to longevity. Relevant variations may include gain-of-function mutations or heterozygous loss-of-function mutations in genes with deleterious effect late in life. Novel biomarkers represented by omics profiles and ambulatory measures to establish the biological aging rate (such as 24-hour glucose 88) will be used in integrated analyses. It has already become feasible to study the integrative personal omics profiles (iPOP), the combination of the genetic, transciptomic, proteomic, metabolomic, and autoantibody profile of individuals 89.

In conclusion, novel methodologies, comprehensively applied to multiple studies of well-phenotyped (middle and highly aged) individuals from long-lived families and large prospective cohort studies, will help to connect human molecular epidemiology and biology in aging research. Ultimately, this will provide leads that can be followed up in animal studies.

## Abbreviations

CHARGE: Cohorts for Heart and Aging Research in Genomic Epidemiology
CNV: copy number variant
FHS: Framingham Heart Study
GEHA: GEnetics of Healthy Ageing
GH: growth hormone
GWAS: genome-wide association study
IDEAL: Integrated research on Developmental determinants of Aging and Longevity
IGF-1: insulin-like growth factor 1
iPOP: integrative personal omics profile
LLFS: Long Life Family Study
LLS: Leiden Longevity Study
LOD: logarithm (base 10) of odds
MRI: magnetic resonance imaging
mTOR: mammalian target of rapamycin
NECS: New England Centenarian Study
NHGRI: National Human Genome Research Institute
NMR: nuclear magnetic resonance
ROH: region of homozygosity
SICS: Southern Italian Centenarian Study
SNP: single nucleotide polymorphism

## Supplementary material

As a service to our authors and readers, this journal provides supporting information supplied by the authors. Such materials are peer reviewed and may be re-organized for online delivery, but are not copy-edited or typeset. Technical support issues arising from supporting information (other than missing files) should be addressed to the authors.
