# Supplementary material for: Identifying the genomic determinants of aging and longevity in human population studies: Progress and challenges
Source: Bioessays. 2013 Feb 19;35(4):386–96. doi: 10.1002/bies.201200148 (PMC3633240; doi:10.1002/bies.201200148)
Supplement: Supplementary file 1 [file bies0035-0386-SD1.pdf]

Supplementary Information

Supplementary Table 1. Identified candidate genes involved in longevity.

Pathway	Analysis	Genes	References
DNA damage signaling and repair	Pathway-based	*EXO1*, *POLB*, *NTLH1*, *RAD23B*, *RAD52*, ***WRN***	[1]
	Single gene-based	*EXO1*	[2]
GH/insulin/IGF-1 signaling	Pathway-based	*AKT1*, *AKT3*, *FOXO3A*, *FOXO4*, *GHRHR*, *GHSR*, *IGF2*, *IGF2R*, *INS*, ***KL***, *PIK3CA*, *SGK1*, *SGK2*, *YWHAG*	[1, 3-5]
	Single gene-based	*FOXO1*, *FOXO3A*, ***IGF1R***, ***SIRT3***	[6-16]
Immune regulation	Pathway-based	*HSF2*	[17]
	Single gene-based	***HSPA1A***, ***HSPA1L***, *HSPA14*, *HSPA1B*, ***IL6***, *TLR4*	[15, 18-22]
Pro/antioxidant	Pathway-based	*GSR*	[1]
	Single gene-based	*GPX1*, ***PON1***, ***SOD2***	[23-26]
Telomere maintenance	Pathway-based	*POT1*	[3]
	Single gene-based	*TERC*, *TERT*	[27, 28]
Lipoprotein metabolism	Single gene based	***APOE***, ***CETP***	[15, 25, 29-33]
RNA editing	Single gene-based	*ADARB1, ADARB2*	[34]
Other	Single gene-based	*A2M*, ***ACE***, *LMNA*	[15, 25, 35-39]

Genes depicted in **bold** were previously reported in the review of Christensen and colleagues [40].

References

1. **Soerensen M, Dato S, Tan Q, Thinggaard M,** et al. 2012. Human longevity and variation in GH/IGF-1/insulin signaling, DNA damage signaling and repair and pro/antioxidant pathway genes: Cross sectional and longitudinal studies. *Exp Gerontol* **47**: 379-87.

2. **Nebel A, Flachsbart F, Till A, Caliebe A,** et al. 2009. A functional EXO1 promoter variant is associated with prolonged life expectancy in centenarians. *Mech Ageing Dev* **130**: 691-9.

3. **Deelen J, Uh HW, Monajemi R, van Heemst D,** et al. 2013. Gene set analysis of GWAS data for human longevity highlights the relevance of the insulin/IGF-1 signaling and telomere maintenance pathways. *Age (Dordr)* **35**: 235-49.

4. **Pawlikowska L, Hu D, Huntsman S, Sung A,** et al. 2009. Association of common genetic variation in the insulin/IGF1 signaling pathway with human longevity. *Aging Cell* **8**: 460-72.

5. **van Heemst D, Beekman M, Mooijaart SP, Heijmans BT,** et al. 2005. Reduced insulin/IGF-1 signalling and human longevity. *Aging Cell* **4**: 79-85.

6. **Anselmi CV, Malovini A, Roncarati R, Novelli V,** et al. 2009. Association of the FOXO3A locus with extreme longevity in a southern Italian centenarian study. *Rejuvenation Res* **12**: 95-104.

7. **Flachsbart F, Caliebe A, Kleindorp R, Blanche H,** et al. 2009. Association of FOXO3A variation with human longevity confirmed in German centenarians. *Proc Natl Acad Sci U S A* **106**: 2700-5.

8. **Kleindorp R, Flachsbart F, Puca AA, Malovini A,** et al. 2011. Candidate gene study of FOXO1, FOXO4, and FOXO6 reveals no association with human longevity in Germans. *Aging Cell* **10**: 622-8.

9. **Kuningas M, Magi R, Westendorp RG, Slagboom PE,** et al. 2007. Haplotypes in the human Foxo1a and Foxo3a genes; impact on disease and mortality at old age. *Eur J Hum Genet* **15**: 294-301.

10. **Kuningas M, Putters M, Westendorp RG, Slagboom PE,** et al. 2007. SIRT1 gene, age-related diseases, and mortality: the Leiden 85-plus study. *J Gerontol A Biol Sci Med Sci* **62**: 960-5.

11. **Lescai F, Blanche H, Nebel A, Beekman M,** et al. 2009. Human longevity and 11p15.5: a study in 1321 centenarians. *Eur J Hum Genet* **17**: 1515-9.

12. **Li Y, Wang WJ, Cao H, Lu J,** et al. 2009. Genetic association of FOXO1A and FOXO3A with longevity trait in Han Chinese populations. *Hum Mol Genet* **18**: 4897-904.

13. **Nygaard M, Soerensen M, Flachsbart F, Mengel-From J,** et al. 2012. AKT1 fails to replicate as a longevity-associated gene in Danish and German nonagenarians and centenarians. *Eur J Hum Genet* in press DOI: 10.1038/ejhg.2012.196.

14. **Soerensen M, Dato S, Christensen K, McGue M,** et al. 2010. Replication of an Association of Variation in the FOXO3A Gene with Human Longevity Using Both Case-control and Longitudinal Data. *Aging Cell* **9**: 1010-7.

15. **Soerensen M, Dato S, Tan Q, Thinggaard M,** et al. 2012. Evidence from case-control and longitudinal studies supports associations of genetic variation in APOE, CETP, and IL6 with human longevity. *Age (Dordr)* in press DOI: 10.1007/s11357-011-9373-7.

16. **Willcox BJ, Donlon TA, He Q, Chen R,** et al. 2008. FOXO3A genotype is strongly associated with human longevity. *Proc Natl Acad Sci U S A* **105**: 13987-92.

17. **Broer L, Demerath EW, Garcia ME, Homuth G,** et al. 2012. Association of heat shock proteins with all-cause mortality. *Age (Dordr)* in press DOI: 10.1007/s11357-012-9417-7.

18. **Capurso C, Solfrizzi V, D'introno A, Colacicco AM,** et al. 2007. Interleukin 6 variable number of tandem repeats (VNTR) gene polymorphism in centenarians. *Ann Hum Genet* **71**: 843-8.

19. **Di Bona D, Vasto S, Capurso C, Christiansen L,** et al. 2009. Effect of interleukin-6 polymorphisms on human longevity: a systematic review and meta-analysis. *Ageing Res Rev* **8**: 36-42.

20. **Li J, Niu W, Qi Y, Mayila W,** et al. 2009. Interactive association of heat shock protein 70 genes variants with natural longevity in Xinjiang Hetian Uygur ethnicity. *Transl Res* **154**: 257-64.

21. **Nebel A, Flachsbart F, Schafer A, Nothnagel M,** et al. 2007. Role of the toll-like receptor 4 polymorphism Asp299Gly in longevity and myocardial infarction in German men. *Mech Ageing Dev* **128**: 409-11.

22. **Singh R, Kolvraa S, Bross P, Christensen K,** et al. 2010. Anti-inflammatory heat shock protein 70 genes are positively associated with human survival. *Curr Pharm Des* **16**: 796-801.

23. **Caliebe A, Kleindorp R, Blanche H, Christiansen L,** et al. 2010. No or only population-specific effect of PON1 on human longevity: a comprehensive meta-analysis. *Ageing Res Rev* **9**: 238-44.

24. **Lescai F, Marchegiani F, Franceschi C**. 2009. PON1 is a longevity gene: results of a meta-analysis. *Ageing Res Rev* **8**: 277-84.

25. **Novelli V, Viviani AC, Roncarati R, Guffanti G,** et al. 2008. Lack of replication of genetic associations with human longevity. *Biogerontology* **9**: 85-92.

26. **Soerensen M, Christensen K, Stevnsner T, Christiansen L**. 2009. The Mn-superoxide dismutase single nucleotide polymorphism rs4880 and the glutathione peroxidase 1 single nucleotide polymorphism rs1050450 are associated with aging and longevity in the oldest old. *Mech Ageing Dev* **130**: 308-14.

27. **Atzmon G, Cho M, Cawthon RM, Budagov T,** et al. 2010. Evolution in health and medicine Sackler colloquium: Genetic variation in human telomerase is associated with telomere length in Ashkenazi centenarians. *Proc Natl Acad Sci U S A* **107 Suppl 1**: 1710-7.

28. **Soerensen M, Thinggaard M, Nygaard M, Dato S,** et al. 2012. Genetic variation in TERT and TERC and human leukocyte telomere length and longevity: a cross-sectional and longitudinal analysis. *Aging Cell* **11**: 223-7.

29. **Jacobsen R, Martinussen T, Christiansen L, Jeune B,** et al. 2010. Increased effect of the ApoE gene on survival at advanced age in healthy and long-lived Danes: two nationwide cohort studies. *Aging Cell* **9**: 1004-9.

30. **Maruszak A, Peplonska B, Safranow K, Chodakowska-Zebrowska M,** et al. 2012. TOMM40 rs10524523 polymorphism's role in late-onset Alzheimer's disease and in longevity. *J Alzheimers Dis* **28**: 309-22.

31. **McKay GJ, Silvestri G, Chakravarthy U, Dasari S,** et al. 2011. Variations in apolipoprotein E frequency with age in a pooled analysis of a large group of older people. *Am J Epidemiol* **173**: 1357-64.

32. **Napolioni V, Gianni P, Carpi FM, Predazzi IM,** et al. 2011. APOE haplotypes are associated with human longevity in a Central Italy population: evidence for epistasis with HP 1/2 polymorphism. *Clin Chim Acta* **412**: 1821-4.

33. **Schachter F, Faure-Delanef L, Guenot F, Rouger H,** et al. 1994. Genetic associations with human longevity at the APOE and ACE loci. *Nat Genet* **6**: 29-32.

34. **Sebastiani P, Montano M, Puca A, Solovieff N,** et al. 2009. RNA editing genes associated with extreme old age in humans and with lifespan in C. elegans. *PLoS One* **4**: e8210.

35. **Conneely KN, Capell BC, Erdos MR, Sebastiani P,** et al. 2012. Human longevity and common variations in the LMNA gene: a meta-analysis. *Aging Cell* **11**: 475-81.

36. **Flachsbart F, Caliebe A, Nothnagel M, Kleindorp R,** et al. 2010. Depletion of potential A2M risk haplotype for Alzheimer's disease in long-lived individuals. *Eur J Hum Genet* **18**: 59-61.

37. **Khabour OF, Abdelhalim ES, bu-Wardeh A**. 2009. Association between SOD2 T-9C and MTHFR C677T polymorphisms and longevity: a study in Jordanian population. *BMC Geriatr* **9**: 57.

38. **Yang JK, Gong YY, Xie L, Lian SG,** et al. 2009. Lack of genetic association between the angiotensin-converting enzyme gene insertion/deletion polymorphism and longevity in a Han Chinese population. *J Renin Angiotensin Aldosterone Syst* **10**: 115-8.

39. **Zajc Petranović M, Skarić-Jurić T, Smolej Narančić N, Tomas Z,** et al. 2011. Angiotensin-converting enzyme deletion allele is beneficial for the longevity of Europeans. *Age (Dordr)* **34**: 583-95.

40. **Christensen K, Johnson TE, Vaupel JW**. 2006. The quest for genetic determinants of human longevity: challenges and insights. *Nat Rev Genet* **7**: 436-48.
